# Optimization of Sustainable Single-Cell Oil Production by Rhodotorula mucilaginosa (BT59) from Grape Pomace and Its Functional Characterization

**DOI:** 10.1021/acsomega.5c01720

**Published:** 2025-05-30

**Authors:** Emel Yücel, Tuncay Gümüş, Deniz D. Altan Kamer, Gülce B. Kaynarca, Murat Taşan

**Affiliations:** 1 Department of Food Engineering, Faculty of Agriculture, 162334 Tekirdag Namik Kemal University, Tekirdag 59030, Turkey; 2 Department of Food Engineering, Faculty of Engineering, 187469 Kirklareli University, Kirklareli 39100, Turkey

## Abstract

The current study reports the optimization of single-cell oil (SCO) production from Rhodotorula mucilaginosa, a promising yeast for industrial applications, with grape pomace as a cost-effective carbon source. Fermentation duration, carbon amount, and pH parameters were used to determine optimum conditions for the production of SCO with the highest yield (5.81 days fermentation, 6.20% carbon amount, and pH 4.33), the highest saturated fatty acid (SFA) content (8 days, 8.32% carbon amount, and pH 4.44), and the highest unsaturated fatty acid content (8 days, 2% carbon amount, and pH 4.47). Oleic acid was identified as the dominant fatty acid (FA) in all samples, accounting for 58–76% of the total FAs, followed by palmitic acid, an SFA, at 10–14%. The amount of bioactive components of SCOs varied depending on the fermentation conditions. The highest total phenolic matter (TPC) and antioxidant activity observed were 659.40 mg GAE (gallic acid equivalent)/kg extract, 24.04 mg/L DPPH· scavenging activities (IC_50_), and 30.43 (μmol Trolox/kg) (TAC value). Additionally, the SCOs exhibited notable antimicrobial activity, particularly against Salmonella enterica subsp. R. mucilaginosa can synthesize SCOs from grape pomace that contains diverse fatty acids, such as oleic, palmitic, γ-linolenic, and docosahexaenoic acids. This microorganism’s ability to generate elevated ratios of desirable fatty acids under particular conditions renders the resultant oils compositionally akin to vegetable oils with analogous fatty acids, such as cocoa butter, palm oil, or olive oil. Moreover, microbial oils have benefits including sustainability, less reliance on agricultural land, and cost-effective use of agricultural waste, thereby establishing these single-cell oils as viable alternatives to conventional vegetable oils.

## Graphical abstract

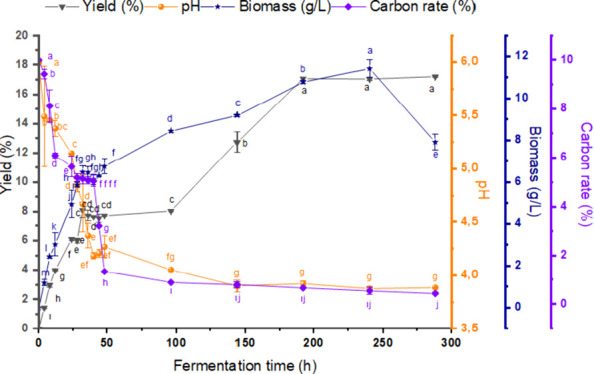

## Introduction

The ever increasing human population and the reduction of agricultural land entail a need for the exploration of alternative sources for lipids and fatty acids.^1^ Microbial oil production has become a prominent focus of research driven by the need for sustainable industrial substitutes for animal- and plant-derived lipids. Microbial oils, synthesized by lipid-accumulating microorganisms, present a potential means by which to supplement conventional fats and oils. Their composition resembles that of prevalent vegetable oils, including soybean, rapeseed, sunflower, and palm oils, making them suitable for industrial applications.^2^

Microbial lipids present a scalable alternative to traditional oils due to the short life cycle of the microorganisms, minimal labor requirements, and resilience to seasonal and climatic changes.^3^ Such lipids not only can enhance sustainability but also have health benefits by providing omega-3 fats that can be used in nutritional supplements, pharmaceuticals, and other consumer products. A growing commercial need for polyunsaturated fatty acids (PUFAs) can be met by single-cell oils (SCO), especially those high in oleic acid, γ-linolenic acid (GLA), arachidonic acid (ARA), eicosapentaenoic acid (EPA), and docosahexaenoic acid (DHA).^4^ Current methods of vegetable oil production encounter sustainability challenges such as land use, deforestation, and competition for food resources. The high levels of global palm oil production and unsustainable farming practices such as excessive pesticide usage and genetically modified crops can lead to deforestation, habitat destruction, and pollution, posing environmental and health risks.^5–11^ Microbial oils offer an environmentally sustainable alternative that is not impacted by these concerns.^5^ However, the high production costs of microbial oils currently limit their economic competitiveness.^7,8^ Using agri-food waste as a carbon source may help reduce the cost of production and advance the use of microbial lipids as a sustainable alternative that can support both environmental goals and human health.

Microbial lipids, particularly those produced by red oil yeast species like Rhodotorula mucilaginosa, are characterized by their sustainable production and rich bioactive profile, including valuable carotenoids.^12^ R. mucilaginosa can grow on a wide range of substrates, including agricultural waste, which reduces manufacturing costs and their environmental impact. Lipid accumulation is affected by variables, including the carbon/nitrogen (C/N) ratio, pH, and substrate composition. Previous studies have reported significant lipid yields in media utilizing economical carbon sources such as sugar cane molasses (39.5%),^13^ corn liquor (37.6%),^14^ wheat straw hydrolysate (1–15%),^15^ waste glycerol (5.36–9.50%),^16^ wheat bran (32.7%),^17^ and bagasse hydrolysate (20.4%).^18^ Modifications in the composition of the substrate can change both the yield and the fatty acid profile of microbial lipids.^5,12–15,19^

All living macro- and microorganisms synthesize lipids for essential structural and functional purposes, including the formation of permeable membrane bilayers for cells and organelles. Nevertheless, a limited number of microbes can amass cellular lipids in quantities beyond 20% or even reaching 80% of their cellular mass.^20^ *Rhodococcus* sp. and Rhodococcus opacus are the most prevalent bacteria utilized in microbial lipid production, alongside *Cryptococcus*, *Cunninghamella*, and *Mortierella.* ^21,22^ The scaled production of essential fatty acids produced by various molds has recently become a subject of consideration.^23^ Examples of certain mold fungi include Rhizopus arrhizus, Mortierella isabellina, and Claviceps purpurea.^24^ Rhodotorula gracilis, Cryptococcus curvatus,^22^ Candida curvata, Lipomyces lipofer, and Rhodosporidium toruloides are the principal yeasts utilized in lipid production.^24^ Bacteria accumulate only complex lipids, which complicates fat production, despite their higher growth rate than other microorganisms.^25^ Molds often exhibit slower growth than yeasts; however, the multi-PUFA content of the SCO oils extracted is superior to that of the oils derived from yeasts. Nonetheless, molds present several challenges, including sluggish development according to their filamentous architecture and diminished biomass output from carbon sources.^22^ So far, various research have been undertaken on yeasts due to their remarkable capability to accumulate substantial amounts of intracellular oil, their development rates, and the similarity of their TAG fractions with plant oils.^26^

Agricultural wastes, such as grape pomace, are valuable substrates for producing high-value biotechnological products. Grape pomace, a byproduct of grape pomace, wine, and molasses production, constitutes 15–25% of the content of processed grapes. Annually, around 13 million tons of grape pomace are generated worldwide, but a limited portion is effectively recycled or repurposed, resulting in significant environmental issues.^27^ Fresh grape pomace, containing 15–20% fermentable carbohydrates, has been utilized for the production of gellan gum,^28^ enzymes,^29^ organic acids,^30^ bioethanol,^31^ and biopolymers^32^ via biotechnological methods and is also appropriate for SCO production, highlighting its use as a sustainable resource. To the best of our knowledge, the use of grape molasses as a source of carbon for the generation of SCO has not been reported to date.

The current study utilized the indigenous yeast isolate R. mucilaginosa (BT59), which was previously isolated and characterized by us. Fermentation conditions were optimized to obtain SCO with varying qualities and specific oil compositions, thereby enabling the production of SCO with a fatty acid composition tailored to the desired industrial application. Such an approach can not only enhance flexibility in production processes but also contribute significantly to solutions tailored to industrial needs by enabling a more efficient use of resources. Previous studies have demonstrated that both the initial pH and carbon source concentration are among the most influential factors affecting microbial lipid accumulation, as they directly impact metabolic fluxes and lipid biosynthesis pathways.^33,34^ The parameters of fermentation, such as the initial pH and substrate concentration, were optimized to enhance the yield of SCO with specific fatty acid profiles. The resulting SCO was analyzed for functional properties, fatty acid and organic acid profiles, and total carotenoid content and composition, along with antioxidant and antimicrobial activities.

## Materials and Methods

### Material

The strain Rhodotorula mucilaginosa (BT59) was isolated, and its molecular characterization has been reported in our previous studies.^35,36^ The grape pomace used as the substrate was obtained as dried grape juice waste from Tekirda Viticulture Research Institute (Tekirdag, Turkey). The grape pomace was stored at −18 °C until its use.

### Microorganism and Culture Medium

Rhodotorula mucilaginosa was maintained on yeast extract peptone dextrose (YPD) slant agar containing 10 g/L yeast extract, 10 g/L peptone, 20 g/L glucose, and 20 g/L agar at 4 °C.^37^

### Determination of Fermentation Kinetics

The kinetic model of cell growth and sugar conversion in the trials for SCO production was investigated over a 12 day fermentation period using the Rhodotorula mucilaginosa BT59 isolate. Data on biomass concentration versus time was collected during the fermentation period.^38^ The changes in pH, substrate, biomass, single-cell oil content, and carbon content were assessed at specific time points (0, 4, 8, 12, 24, 28, 32, 36, 40, 44, 48, 96, 144, 192, 240, and 248 h) during the fermentation process.

### Kinetic Model of Cell Growth

Sterile flasks containing the medium were inoculated with the isolate, and the flasks were incubated on a shaker (Shaker KS 130 basic, IKA-Werke GmbH & Co. KG, Germany) at 200 rpm throughout the fermentation process. Aerobic conditions were established by ensuring oxygen availability for cell growth.

To evaluate the effect of sugars on microbial growth, the relationship between the initial sugar concentration and specific growth rate of the biomass was examined. Biomass concentration was measured, and specific growth rates were calculated to assess their variation with the sugar concentration. Substrate inhibition was analyzed to determine specific growth rates at different sugar concentrations.^38^

The sugar conversion rate in the fermentation kinetics was calculated according to the following equation:

$$\mathrm{sugar}\;\mathrm{conversion}(\%)=\frac{\left(S_{m}-S_{e}\right)}{S_{m}}*100$$ 1

where *S*_m_ is the amount of sugar in the inoculated medium and *S*_e_ is the amount of sugar at the end of fermentation. Five milliliters of liquid was taken on different days of fermentation and centrifuged at 10,000 rpm for 10 min; the supernatant was separated, and the precipitated biomass was dried. The biomass was calculated in g/L.^39^

### Single-Cell Oil Production and Mathematical Modeling

The yeast culture was transferred to a YPD medium and grown to a density equivalent to 0.5 McFarland (1.5 × 10^8^ CFU/mL) at 25 °C with shaking at 200 rpm for 24 h. Subsequently, 10% (v/v) of the inoculum was inoculated into the production medium. SCO production was carried out by aerobic fermentation in a sterile medium with continuous shaking, containing grape pomace as a carbon source, ammonium sulfate as a nitrogen source, and other trace elements. The medium for lipid production was adjusted to contain 2.5 g/L Na_2_HPO_4_, 7.0 g/L KH_2_PO_4_, 1.5 g/L MgSO_4_.7H_2_O, 0.2 g/L yeast extract, and 0.2 g/L (NH_4_)_2_SO_4._ ^40^ Preliminary experiments carried out with 15, 10, and 4% carbon contents indicated that the optimal amount of carbon was 10% since the oil yield did not increase any further when the carbon amount was increased to 15%; additionally, the use of a lower amount of grape pomace was more economical. Fermentation conditions were set at 30 °C with shaking at 200 rpm. The air flow rate was set at 1 min/L.^41^

Further preliminary experiments were carried out to optimize the fermentation rate (both maximal and minimal points) with varying substrate amounts (2–10%), pH (3.5–6), and duration of fermentation (2–8 days). Next, the central composite experimental design was used with the response surface method of the Design Expert version 13 (State-Ease Inc., Minneapolis, MN, USA) and the most important parameters that provided the highest SCO content were determined. The central composite design generated by the Design Expert software was used to identify the optimum model for the designated variables based on the coefficient of variation and *R* ^2^ values. The oil yield and the SFA and unsaturated fatty acid (USFA) contents were selected as the dependent variables, while time, substrate amount, and pH were used as independent variables in the statistical and mathematical modeling. The most effective parameters were determined by preliminary experiments. The randomized combinations determined by the experimental design are given in Table 1.

**Table 1:** Central Composite Experimental Design and Responses^a^

	**independent variables**			**responses**		
**runs**	**fermentation duration (days)**	**carbon content (%)**	**pH**	**yield**(g/L)	**saturated fatty acid (%)**	**unsaturated fatty acid (%)**
1	5.00	11.26	4.75	13.07	17.84	82.16
2	5.00	6.00	3.10	20.32	18.08	81.93
3	2.00	10.00	3.50	14.22	16.68	83.32
4	8.00	10.00	3.50	20.05	19.40	80.60
5	2.00	2.00	3.50	11.78	18.12	82.61
6	5.00	6.00	4.75	23.39	18.48	82.63
7	5.00	6.00	4.75	25.88	19.17	81.55
8	5.00	6.00	4.75	24.65	18.48	83.73
9	2.00	10.00	6.00	8.28	15.83	84.17
10	5.00	0.73	4.75	11.78	12.92	87.08
11	5.00	6.00	4.75	23.90	19.77	81.55
12	8.00	10.00	6.00	11.18	18.41	81.58
13	8.00	2.00	3.50	16.19	14.30	86.71
14	5.00	6.00	6.39	11.54	20.06	81.55
15	5.00	6.00	4.75	21.98	18.37	81.63
16	8.94	6.00	4.75	16.59	19.70	80.30
17	8.00	2.00	6.00	13.99	15.89	85.70
18	2.00	2.00	6.00	14.19	18.08	81.92
19	1.05	6.00	4.75	ND	ND	ND
20	5.00	6.00	4.75	22.19	18.81	83.14

a ND: not detected.

### Single-Cell Oil Extraction and Yield

Lipids were extracted from 1 g of dry biomass using 10 mL of chloroform–methanol (2:1, v/v) over a period of 48 h at room temperature (25 °C).

The final homogenate was filtered and washed with 10 mL of a 0.9% NaCl solution.

The sample was subsequently centrifuged at 2000 rpm for 5 min at room temperature (25 °C) to isolate the lipid-containing chloroform phase. The chloroform was evaporated using a vacuum rotary evaporator at a temperature of 50–55 °C.^42^ The lipid content was determined according to eq 2.

$$\mathrm{yields}(\frac{g}{g}\mathrm{dry}\;\mathrm{weight})=\frac{\mathrm{raw}\;\mathrm{fat}\;\mathrm{mass}(g)}{\mathrm{dry}\;\mathrm{biomass}(g)}$$ 2

### GC-FID Analysis

GC-FID was used to identify the free fatty acids produced in the TAG form. For this, the free fatty acids in the oil extracts were converted to volatile methyl esters by transesterification.^43^ For fatty acids to be detected by GC-FID, they must be transformed from their free fatty acid form to volatile derivatives. Consequently, the oil extracts undergo transesterification to yield methyl esters. During the derivatization process, 100 mg of oil extract was vortexed at ambient temperature until fully dissolved in 10 mL of hexane. Subsequently, 0.5 mL of 2 N KOH (and MeOH) was incorporated into the solution and agitated using a vortex apparatus for 30 s. The supernatant will be stored in the darkness for 1–2 h until it becomes transparent. After a waiting period, the upper phase (hexane) from the binary phase will be isolated and passed to the GC vial.^43^

The examination of volatile fatty acid derivatives was conducted using a SHIMADZU 2010 Gas Chromatography (GC) apparatus. The gas chromatograph was utilized alongside a flame ionization detector (FID) for analytical purposes. The TR-CN100 capillary column (0.25 × 100 × 0.2 mm) was utilized for fatty acid analysis. The inlet temperature was established at 250 °C. The flow rate was established at 30 mL/min with helium as the carrier gas. The oven temperature will initiate at 100 °C, escalating to 240 °C at a rate of 3 °C/min, and will be sustained at 240 °C for 10 min, culminating in a total length of 60 min.

### Free Fatty Acid Content and Peroxide Value

Free fatty acid content of SCO samples was determined as % oleic acid according to the AOCS Ca 5a-40 method. The peroxide value of the oil samples was determined according to AOCS Cd 8-53.^44^

### Bioactive Compound Analysis

The dried biomass samples and SCO were pulverized for extraction and extracted with 80% methanol at a ratio of 1:6 for 6 h at 200 rpm in a shaking incubator (Shaker KS 130 basic, IKA-Werke GmbH & Co. KG, Germany).^45^

Antioxidant activity was evaluated using both 1,1-diphenyl-2-picrylhydrazyl (DPPH) and cupric reducing antioxidant activity (CUPRAC) methods. Methanolic extracts of the solutions were mixed with 600 μL of 1 mM DPPH (1,1-diphenyl-2-picrylhydrazyl) methanolic solution to a final volume of 6000 μL followed by 30 min of incubation in the dark. The absorbance of the solution was measured at 517 nm, and the IC_50_ value for radical scavenging activity was calculated (mg L^–1^). The corresponding blank readings were also taken, and percent inhibition was then calculated as follows:

$$\mathrm{inhibition}(\%)=\frac{A_{\mathrm{blank}}-A_{\mathrm{sample}}}{A_{\mathrm{blank}}}*100$$ 3

A blank represents the absorbance of the control reaction, which includes all reagents except the test compound, while the example denotes the absorbance of the test compound. The IC50 value, representing the concentration of the sample necessary for 50% scavenging of the DPPH free radical, was derived from the plot of percent scavenging against the concentration. Each determination was conducted in triplicate, and the mean IC50 value was computed.^46^

For the CUPRAC assay, 500 μL of the sample solution was mixed with 600 μL of distilled water, 1000 μL of 0.0075 M neocuproine (2,9-dimethyl-1,10-phenanthroline) in ethanol, and 1000 μL of 0.01 M copper II chloride. The mixture was incubated in the dark for an hour at 25 °C before measuring the absorbance at 450 nm.^47^ Trolox equivalent antioxidant activity (TEAC) is the millimolar concentration of a Trolox solution that exhibits antioxidant activity equivalent to that of a 1.0 mM solution of the analyzed chemical. TEAC coefficients are calculated by dividing the measured molar absorptivity of each antioxidant by the molar absorptivity of Trolox (εTR = 1.58 × 10^4^ L/mol/cm at specified conditions, with an optical cuvette thickness of 1 cm). The antioxidant activity was expressed in μmol of TE/g of dry extract. The total antioxidant activity (TAC) of the sample was determined as follows:

$$\mathrm{TAC}(\frac{\mu \mathrm{mol}\;\mathrm{TE}}{g\;\mathrm{dry}\;\mathrm{extract}})=(\frac{\mathrm{absorbance}}{\varepsilon R}){*}^{\;}\frac{\mathrm{total}\;\mathrm{volume}}{\mathrm{extract}\;\mathrm{volume}}{*}^{\;}(\mathrm{dilution}\;\mathrm{factor}){*}^{\;}\frac{\mathrm{extract}\;\mathrm{volume}}{\mathrm{dry}\;\mathrm{weight}\;\mathrm{of}\;\mathrm{sample}}$$ 4

The total phenolic content was determined with the Folin–Ciocalteu reagent in an alkaline solution. The resulting color was measured by spectrophotometry (Shimadzu 02910, Tokyo, Japan), and the results were calculated as mg GAE/kg using gallic acid as the standard.^48^ The standard curve was created using the following procedures: a series of gallic acid solution concentrations (0.4, 0.8, 1.2, 1.6, 2.0, and 2.5 mg/L) have been prepared. The equation of the standard curve was obtained as follows:

$$Y=0.0542X+0.0015(R^{2}=0.9785)$$ 5

The samples (40 μL) were diluted 6-fold with pure water, mixed with 200 μL of Folin–Ciocalteu reagent, and incubated for 2 min. Six hundred microliters of this solution was mixed with 75 g L^–1^ Na_2_CO_3_ and 760 μL of distilled water by vortexing followed by measurement of the absorbance at 760 nm. The measurements were taken quickly after 1 h of incubation in the dark.^48^

The total carotenoid content was assessed using the methodology established by Wang et al. (2008). To this end, 50 mL of a hexane–acetone–ethanol (v/v; 50:25:25) mixture was combined with a 5 g powdered sample and extracted at 200 rpm for 10 min at ambient temperature. The combination was centrifuged at 6500 rpm for 5 min at 4 °C, after which the supernatant will be collected and diluted to 50 mL with the extraction solution^49,50^ The absorbance of the extracts was quantified at a wavelength of 450 nm, and results were expressed as mg/100 g β-carotene utilizing the formula provided below (eq 6).

$$\mathrm{total}\;\mathrm{carotenoid}\;\mathrm{amount}=\frac{\mathrm{absorbance}{*}^{\;}\mathrm{Sf}{*}^{\;}10}{\frac{E1}{2}}{*}^{\;}100$$ 6

where E1/2 is the extinction coefficient: 2505 and Sf is the dilution factor.

The disk diffusion method was used to determine the antimicrobial properties of the samples. Microbial cultures (Staphylococcus aureus ATCC 25923, Listeria monocytogenes ATCC 7644, and Salmonella enterica subs. ATCC 13076, and Escherichia coli O157:H7 ATCC 33150) obtained after 18–24 h were inoculated on nutrient agar plates (standardized inoculum: 1–2 × 10^7^ CFU/mL, 0.5 McFarland Standard). Sterile 6 mm diameter disks were placed on the agar, and 20 μL of each tested SCO extract was applied to the disks. Disks containing extract alcohol were used as a positive control. The plates were incubated at 37 °C for 18–24 h and the inhibition zone diameters (in mm) were measured to evaluate the antimicrobial effects of the samples.^51^

### Data Analysis

All experiments were repeated three times. The significance level was set at *p* < 0.05. The data were analyzed statistically using Duncan’s multiple range tests with SPSS 17.0 for Windows (SPSS INC., Chicago, IL, USA). The eigenvector Solo 8.7 program (WA, USA) was run, and the principal component analysis (PCA) toolbox was used for PCA analysis. Autoscaling and mean center preprocessing methods were applied to obtain the highest discrimination rate between the samples. Principal components with root mean squared error of calibration (RMSEC) and root mean squared error of verification (RMSECV) values were selected, and the PCA model was built using the cross-validation method (blinds) for each PCA model. All samples were analyzed in duplicate.

One-way analysis of variance (ANOVA) was used to assess the statistical significance of the proposed models. The precision of the models was assessed using the Design-Expert software to generate *R* ^2^ coefficients, adjusted *R* ^2^ coefficients, and lack of fit parameters. The quadratic model (eq 7) was used to represent the fitted response values.

$$Y=\beta_{0}+\beta_{1}A+\beta_{2}B+\beta_{3}C+\beta_{12}AB+\beta_{13}AC+\beta_{23}BC+\beta_{11}A^{2}+\beta_{22}B^{2}+\beta_{33}C^{2}$$ 7

where *Y* represents the dependent variables such as oil yield, SFA, and USFA content. *X* represents the ratios of the pseudocomponents, and β represents the coefficients of the equation. Optimum formulations were determined by selecting the maximum points separately for oil yield, saturated fat, and unsaturated fat responses.

The Pearson correlation approach in OriginPro 2021 software (OriginLab Corporation, USA) was used to assess potential interactions between total antioxidant activity, total phenolic content, and antibacterial capacity of SCOs.

## Results and Discussion

### Determination of Fermentation Kinetics

The fermentation was carried out in different nutrient media while keeping a constant microbial load (McFarland 0.5), pH of 6.00, and carbon content of 10.00% (Figure 1). Previous studies have reported the impact of varying concentrations of carbon sources on microbial biomass production.^52,53^ The carbon content of 10% was chosen since the synthesis of SCO showed a decrease when the carbon content was increased any further, as also reported previously.^54^

**Figure 1:**
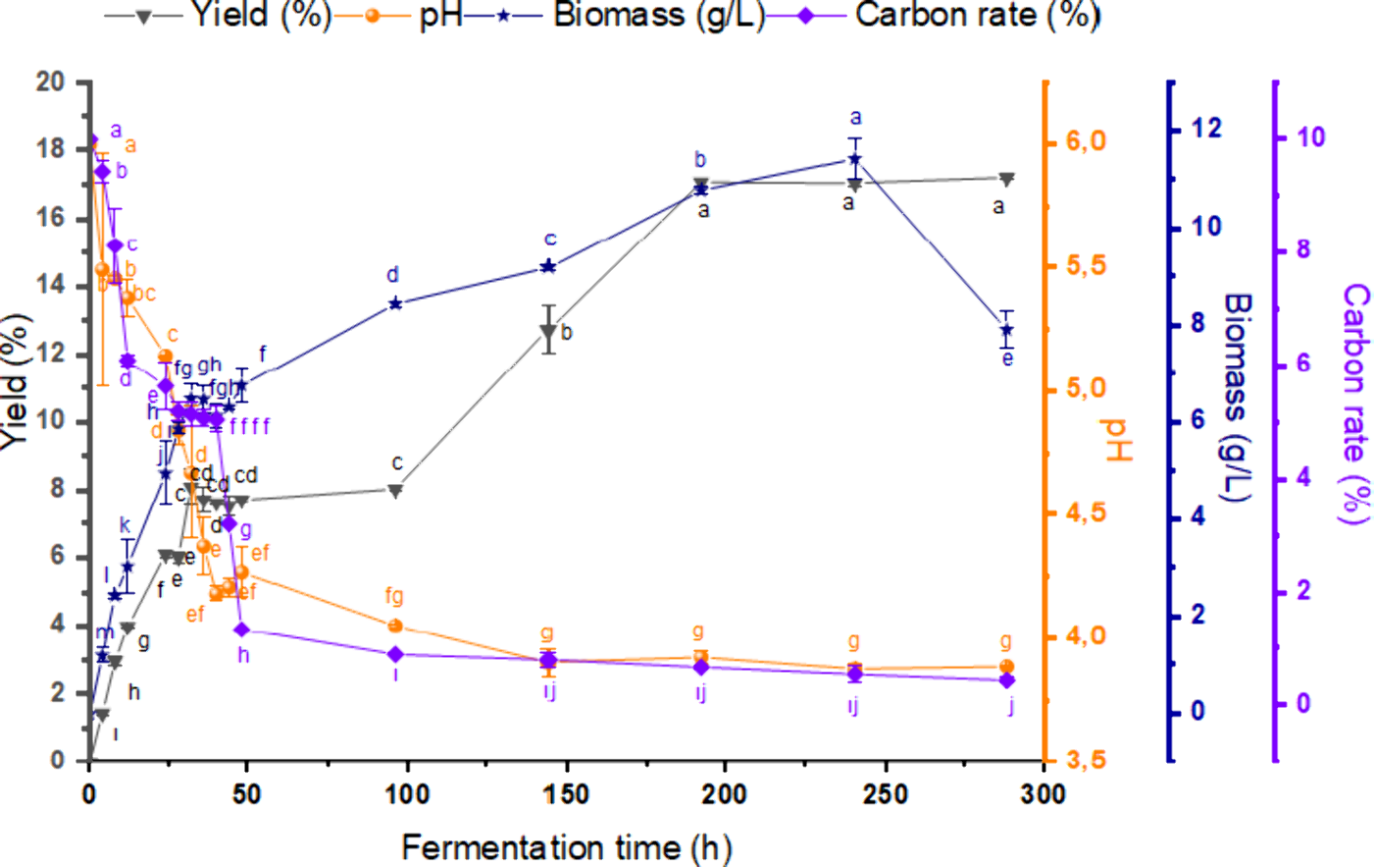
Changes in biomass, oil yield, pH and carbon content during fermentation.^*a–l^ represent statistically difference between samples (*p* < 0.05).

The highest and lowest values of the independent variables (fermentation duration, pH, and carbon amount) that were used for the optimization of SCO production using the Rhodotorula mucilaginosa (BT59) isolate with a kinetic study. Accordingly, the highest % oil yield (17.08 ± 0.11) occurred on the 192 h of fermentation and the highest biomass (11.43 ± 0.41 g/L) increase occurred on the 240 h of fermentation. 90% of the carbon content was used within the first 48 h of fermentation, which coincided with the active growth phase of the microorganism, while biomass and oil production increased after 144 h, supporting the previous literature.^53,55^ The oil yield did not change after the 192 h of fermentation, most likely because the carbon source was exhausted by then,^56^ while the biomass yield showed a significant decrease after the 240 h of fermentation. This suggests the lack of a direct relationship between the biomass yield and oil yield. The pH was found to gradually decrease to 4.37 in the first 36 h period and further to 3.80 at the end of 288 h, which can be attributed to the conversion of the carbon source to organic acids by the cells.^55^ The optimal pH for lipid production is an important parameter to optimize as it can vary for different carbon sources and oleaginous yeast strains.^56^ The increase in oil yield coincident with the decrease in pH played an important role in the selection of the pH change as one of the optimization parameters. Optimization of the fermentation duration is also a critical factor, in terms of cost. Therefore, 192 h, when the SCO yield reached the highest level, was set as the limit for the duration of fermentation. Overall, our kinetic study indicated that the minimum and maximum ranges of the variables for the optimization of SCO yield were 2–10% for carbon content, 3.5–6 for the initial pH, and fermentation duration of 48–192 h.

### Experimental Design and Optimization

Table 1 presents the optimized data for the oil yield, SFA, and USFA contents. The quadratic model was determined to be the most suitable for evaluating the effect of the individual variables and their interactions on the outcome. The ANOVA results for the quadratic model are presented in Table 2. The relationship between the different factors and responses generated with polynomial equations is shown in Table 3.

**Table 2:** Analysis of Variance (ANOVA) Assessment of the Adequacy of the Quadratic Polynomial Fitted Model^a^

	**yield**			**saturated fatty acid (%)**			**unsaturated fatty acid (%)**		
**source**	**sum of squares**	***F*-value**	***p*-value**	**sum of squares**	***F*-value**	***p*-value**	**sum of squares**	***F*-value**	***p*-value**
model	439.26	12.21	0.0002	53.93	17.29	<0.0001	52.66	14.07	<0.0001
*A*				0.0038	0.0049	0.9451	0.0144	0.0154	0.9031
*B*	0.0455	0.006	0.9378	9.46	12.13	0.0037	16.45	17.58	0.0009
*C*	59.67	8.29	0.0129						
*AB*				16.00	20.52	0.0005	21.72	23.21	0.0003
*BC*	28.20	3.92	0.0693						
*A* ^2^									
*B* ^2^	214.47	29.81	0.0001	28.15	36.10	<0.0001	14.42	15.41	0.0015
*C* ^2^	86.91	12.08	0.0041						
lack of fit	82.52	6.25	0.0530	9.46	3.61	0.0854	8.69	1.09	0.4869

a A: fermentation duration (days); B: carbon content; C: pH.

**Table 3:** Regression Coefficients and Correlations of the Model with the Experimental Data^a^

parameter	**equation**	***R*** ^**2**^	**adjusted *R*** ^**2**^	**Adeq precision**
yield	–43.03822 + 5.94407*B* + 21.97928*C* – 0.375500*BC* – 0.348016*B* ^2^ – 2.26857*C* ^2^	0.82	0.76	8.85
saturated oil acid (%)	16.64975 – 0.700386*A* + 1.13994*B* + 0.117844*AB* – 0.125177*B* ^2^	0.83	0.78	11.05
unsaturated oil acid (%)	82.80706 + 0.836831*A* – 0.688076*B* – 0.137316*AB* + 0.089595*B* ^2^	0.80	0.74	11.47

a A: fermentation duration (days); B: carbon content; C: pH.

The significance of the model was assessed with *F* and *p*-values. The models have significant effects on the dependent variables when the F values are high and the p-value is less than 0.05. The model’s suitability is confirmed by the fact that the lack of fit analysis yields a p-value of greater than 0.05, which suggests that there is no significant difference between the experimental data and the model. The duration of fermentation was found to be unrelated to the oil yield, while changes in pH and carbon content were found to be significant. An increase in oil yield was observed as the carbon content approached 6% and the pH value approached 4.5, as illustrated in the 3D graph (Figure 2A). The fact that the highest oil yield of 17.08% obtained in the kinetic study could be increased to 25.88% with the optimization study reveals the efficiency of our approach. The SFA content of SCO was not affected by the pH of the fermentation medium; rather, it increased with an increase in the carbon content and fermentation duration. As the duration of fermentation was increased to 8 days, the proportion of SFA increased as the carbon content approached 8% (Figure 2B). Among the independent variables for USFA content, pH was found to be insignificant, while carbon content and duration of fermentation were found to be significant, similar to our observations with SFA. However, unlike SFA, the USFA content increased as the carbon content was decreased (Figure 2C). The ANOVA data for the regression models provide statistical evidence that the quadratic models were successful in predicting all responses of SCO. Since the maximum points of the dependent variables were found to be quite different from each other, the optimum point was determined separately for each dependent variable.

**Figure 2:**
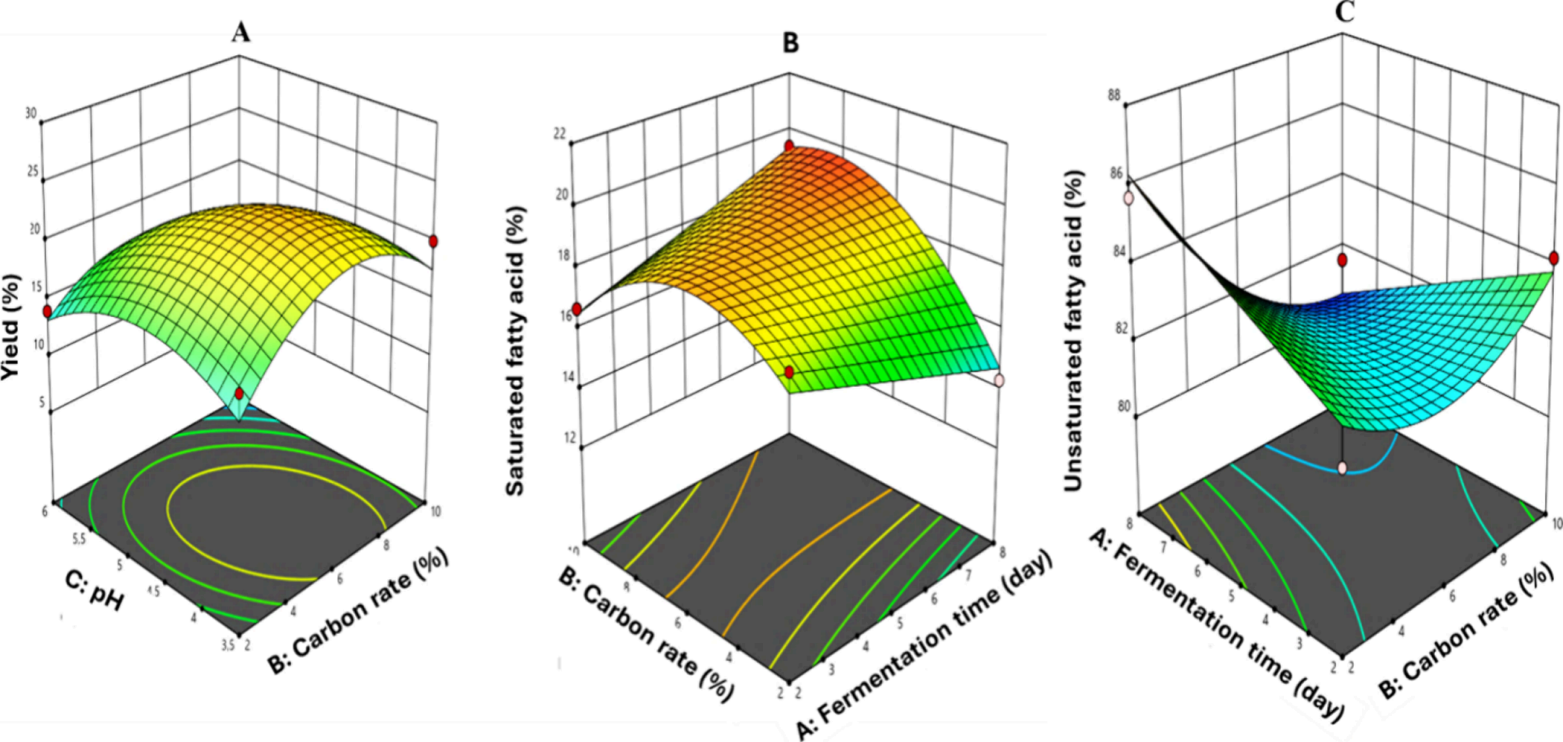
3D surface graph for (A) oil yield, (B) saturated fatty acid content, and (C) unsaturated fatty acid content.

### Determination of Optimal SCO

The optimal SCO production was determined separately for each parameter by considering factors such as oil yield, SFA content, and USFA content. Maximization of each of these parameters was prioritized, while the degree of importance was kept the same (++++) for all factors.

The predicted response of the models in the optimal conditions selected for all responses is also presented in Table 4. When each response was optimized separately, the optimal fermentation duration was calculated as 5.81 days, carbon content was calculated as 6.20%, and pH was calculated as 4.33 for the optimum oil yield. Similarly, for SFA, 8 days with 8.32% carbon content, pH 4.44, and for USFA, 8 days with 2% carbon content, and pH 4.47 were recommended. Desirability was quite high for all of the responses (0.74–0.95). These data suggest that the proposed fermentation conditions can optimize the oil yield as well as the SFA and USFA composition, facilitating the effective production of SCO for diverse applications.

**Table 4:** Optimal Parameters of Single-Cell Oils

		**response**		
parameter		**oil yield (%)**	**unsaturated fatty acids (%)**	**saturated fatty acids (%)**
values	goal	maximum	maximum	maximum
	lower	15.00	80.30	12.91
	upper	25.88	87.08	20.06
selected optimum condition	fermentation duration (days)	5.81	8.00	8.00
	carbon amount (%)	6.20	2.00	8.32
	pH	4.33	4.47	4.44
predicted response		22.99	86.28	19.71
individual desirability		0.74	0.88	0.95

### Fatty Acid Composition of SCO

The fatty acid composition of SCO containing about 18 different fatty acids produced in 20 different trial runs is shown in Tables 5 and 6. These oils contain an average of 18% SFA, 69% monounsaturated FA (MUFA), and 13% polyunsaturated FA (PUFA). The PUFA/SFA ratio of SCOs was found to vary between approximately 0.4 and 1.39,^57^ reported that the PUFA/SFA ratio above 0.4 is critical to ensure an appropriate cholesterol content and to prevent cardiovascular diseases. Our findings and the published literature point toward the health benefits of SCOs.^57^ A study on the chemical and sensory characteristics of Brazilian extra virgin olive oils revealed a PUFA/SFA ratio of 0.7,^45,58^ comparable to our findings. The analysis of the chemical composition and chromatic qualities of oil derived from three aguaje morphotypes (Mauritia flexuosa Lf), extracted using supercritical carbon dioxide, revealed a PUFA/SFA ratio between 0.10 and 0.15.^59^ Our results exceed these values. Consequently, an increased ratio correlates with a more advantageous outcome.^60^

**Table 5:** Fatty Acid Composition of Single-Cell Oils^a^

	experimental runs									
	1	2	3	4	5	6	7	8	9	10
fatty acids (%)	Day:5 Carbon:11.26 pH:4.75	Day:5 Carbon:6 pH:3.10	Day:2 Carbon:10 pH:3.5	Day:8 Carbon:10 pH:3.5	Day:2 Carbon:2 pH:3.5	Day:5 Carbon:6 pH:4.75	Day:5 Carbon:6 pH:4.75	Day:5 Carbon:6 pH:4.75	Day:2 Carbon:10 pH:6	Day:5 Carbon:0.73 pH:3.10
myristic acid (C:14)	0.54	0.59	0.42	0.69	0.45	Nd.	0.66	Nd.	Nd.	Nd.
pentadecanoic acid (C:15)	0.12	0.11	Nd.	0.12	Nd.	Nd.	0.09	Nd.	Nd.	Nd.
palmitic acid (C:16)	12.44	12.87	10.91	13.77	12.64	14.25	13.86	13.36	10.69	10.63
palmitoleic acid (C:16:1)	1.47	1.41	0.94	1.57	0.89	Nd.	1.38	1.77	0.83	0.45
C:17 (heptadecanoic acid)	0.10	0.11	Nd.	0.14	0.11	Nd.	0.11	Nd.	Nd.	Nd.
*cis*-10 heptadecanoic acid (C:17:1)	0.25	0.24	Nd.	0.27	0.21	Nd.	0.25	Nd.	Nd.	Nd.
stearic acid (C:18)	3.18	3.14	3.95	3.34	3.72	3.11	3.29	2.71	3.76	1.77
elaidic acid (C:18n9t)	Nd.	Nd.	Nd.	Nd.	Nd.	Nd.	Nd.	Nd.	Nd.	Nd.
oleic acid (C:18:1n9c)	73.28	75.46	75.30	64.28	75.59	68.11	72.71	74.24	69.73	71.20
linoleic acid (C18:2n6c)	6.13	4.25	5.93	12.97	4.31	14.52	5.87	6.42	12.62	14.44
arachidic acid (C20)	0.19	0.24	Nd.	0.22	0.18	Nd.	0.26	Nd.	0.19	Nd.
γ-linolenic acid (C:18:3n6)	0.68	0.47	0.92	1.33	0.77	Nd.	0.47	0.58	1.00	0.99
behenic acid (C:22)	0.51	0.39	0.41	0.41	0.34	Nd.	0.36	Nd.	0.3718	0.13
tricosanoic acid (C:23)	Nd.	0.07	0.99	0.09	Nd.	Nd.	0.06	Nd.	Nd.	Nd.
lignoceric acid (C:24)	0.75	0.56	Nd.	0.62	0.67	Nd.	0.47	0.42	0.82	0.38
nervonic acid (C:24:1)	0.22	Nd.	Nd.	0.08	Nd.	Nd.	0.05	Nd.	Nd.	Nd.
docosahexaenoic acid (C:22:6n3)	0.14	0.09	0.24	0.10	0.12	Nd.	0.0516	Nd.	Nd.	Nd.

a Nd.: not detected.

**Table 6:** Fatty Acid Composition of Single-Cell Oils^a^

	experimental runs								
	11	12	13	14	15	16	17	18	20
fatty acids (%)	Day:5 Carbon:6 pH:4.75	Day:8 Carbon:10 pH:6	Day:8 Carbon:2 pH:3.5	Day:5 Carbon:6 pH:6.39	Day:5 Carbon:6 pH:4.75	Day:8.94 Carbon:6 pH:4.75	Day:8 Carbon:2 pH:6	Day:2 Carbon:2 pH:6	Day:5 Carbon:6 pH:4.75
myristic acid (C:14)	0.64	0.67	0.36	0.69	0.58	0.72	0.51	0.38	Nd.
pentadecanoic acid (C:15)	Nd.	0.12	0.08	0.10	0.10	0.12	0.09	Nd.	Nd.
palmitic acid (C:16)	14.54	13.21	10.53	14.55	12.84	14.03	12.35	13.01	14.78
palmitoleic acid (C:16:1)	1.28	1.84	1.21	1.33	1.44	1.47	1.38	1.00	0.70
C:17 (heptadecanoic acid)	0.12	0.09	0.06	0.12	0.12	0.13	0.11	0.09	Nd.
*cis*-10 heptadecanoic acid (C:17:1)	0.18	0.25	0.19	0.18	0.11	0.22	0.12	0.22	Nd.
stearic acid (C:18)	3.41	2.98	1.63	3.56	3.37	3.38	1.97	3.57	2.08
elaidic acid (C:18n9t)	Nd.	Nd.	Nd.	Nd.	Nd.	0.047	Nd.	Nd.	Nd.
oleic acid (C:18:1n9c)	68.86	69.12	76.15	66.60	74.74	66.71	72.68	76.33	58.03
linoleic acid (C:18:2n6t)	Nd.	Nd.	Nd.	Nd.	0.10	0.01	0.04	Nd.	Nd.
linoleic acid (C18:2n6c)	8.46	9.47	8.62	10.87	4.64	10.92	9.03	3.64	19.20
arachidic acid (C20)	0.22	0.19	0.06	0.20	0.23	0.24	0.12	0.16	Nd.
γ-linolenic acid (C:18:3n6)	0.71	0.74	0.54	0.93	0.51	0.86	0.65	0.74	0.86
behenic acid (C:22)	0.37	0.39	0.20	0.37	0.40	0.43	0.23	0.21	Nd.
tricosanoic acid (C:23)	Nd.	0.08	Nd.	0.01	0.07	0.10	0.06	Nd.	Nd.
lignoceric acid (C:24)	0.46	0.69	0.38	0.48	0.62	0.49	0.47	0.65	Nd.
nervonic acid (C:24:1)	Nd.	0.09	Nd.	Nd.	0.06	0.05	0.06	Nd.	3.01
docosahexaenoic acid (C:22:6n3)	0.74	0.09	Nd.	0.03	0.03	0.06	0.06	Nd.	Nd.

a Nd.: not detected.

The physicochemical and nutritional properties of oils are also influenced by the types and ratios of FA and their position in the glycerol fraction.^26^ The major FAs obtained in the current study from the SCO samples were oleic acid (C:18:1n9c), palmitic acid (C:16), linoleic acid (C18:2n6c), and stearic acid (C:18). γ-Linolenic acid (C:18:3n6) and palmitoleic acid (C:16:1) were detected to a lesser extent in most of the samples. Depending on the fermentation conditions, the oleic acid content varied between 58.03 and 76.15%. Based on the optimization iterations, our data indicate the presence of higher amounts of unsaturated fatty acids when a lower carbon amount and longer fermentation duration were used. Previous studies have reported an increase in oleic acid content at low pH values.^61^ An evaluation of the SFA composition indicated that the lowest amount of palmitic acid was 10.53%, while the highest amount was 14.78%.

Palm oil has 50% saturated fatty acids (SFAs), predominantly palmitic acid (44%) and a lesser quantity of stearic acid (5%); 40% monounsaturated fatty acids (MUFAs), primarily oleic acid; and 10% polyunsaturated fatty acids (PUFAs), chiefly linoleic acid.^60,62–64^ Numerous research has examined the phenotypic characteristics and genetic factors influencing the fatty acid composition of oil palm.^65^ These investigations have demonstrated considerable heterogeneity in fatty acid composition across E. guineensis populations. The composition of palm oil markedly differs between those of E. guineensis and E. oleifera. The unsaturated fatty acid concentration in E. oleifera palms varies from 47 to 69% for C18:1, 2 to 19% for C18:2, and 0.1 to 1.2% for C18:3.^66,67^ SCOs can be tailored to mimic the profile of palm oil for applications in food inductry, thus eliminating the need for reformulation, hydrogenation, or the use of expensive exotic oil blends.^68^

In the current study, SCO obtained using R. mucilaginosa (BT59) was also evaluated as a potential palm oil alternative. Fermentation conditions optimized for this purpose can provide valuable information on the suitability of SCOs for industrial applications. For example, SCOs can be tailored to mimic the profile of palm oil for applications in food industry, thus eliminating the need for reformulation, hydrogenation, or the use of expensive exotic oil blends.

Our data suggest that a shorter fermentation duration increased the production of palmitic acid in the presence of a higher carbon concentration and low pH value. A higher carbon concentration has previously been reported to be associated with higher palmitic acid production.^69^ Moreover, slightly acidic or neutral pH levels are generally preferred for palmitic acid production.^70^

We also observed that the amount of stearic acid ranged between 1.63 and 3.94%, linoleic acid between 4.25 and 19.20%, and γ-linolenic acid between 0.47 and 1.32%. A previous study has reported that linolenic acid obtained from microbial sources *Chlorococcum* and *Dunaliella* could act as an antibiotics. Furthermore, the SCO samples obtained in the current study contained between 0.03 and 0.14% docosahexaenoic acid (DHA, C:22:6n3) and between 0.38 and 0.81% lignoceric acid (C:24). Depending on the fermentation conditions, the omega-3 fatty acid content (DHA) of SCOs can vary between 0.031 and 0.141%.^71^ In the SCOs generated in the current study, the γ-linolenic acid content varied between 0.473 and 1.328%, while the linoleic acid content (both omega-6 fatty acids) varied between 4.254 and 19.202%, depending on the fermentation conditions.

### Principal Component Analysis (PCA)

A PCA shows how the samples and variables are distributed in two principal component spaces (PC1 and PC2), which can also explain most of the total variance in the data. Each point on the graph represents a sample, while the direction of the vectors shows the relationship of the variables with the samples.

SCOs were evaluated at 20 different test points with varying compositions and ratios under different fermentation conditions in the optimization runs. The PCA (Figure 3) addressed whether there was any similarity in the composition of the obtained SCOs. We observed that the PC1 axis explained 87.15% of the variance, while the PC2 axis explained 10.28% of the variance. PC1 and PC2 together could account for 97.43% of the total variance, while the RMSEC/RMSECV values were 0.246/0.394, respectively.

**Figure 3:**
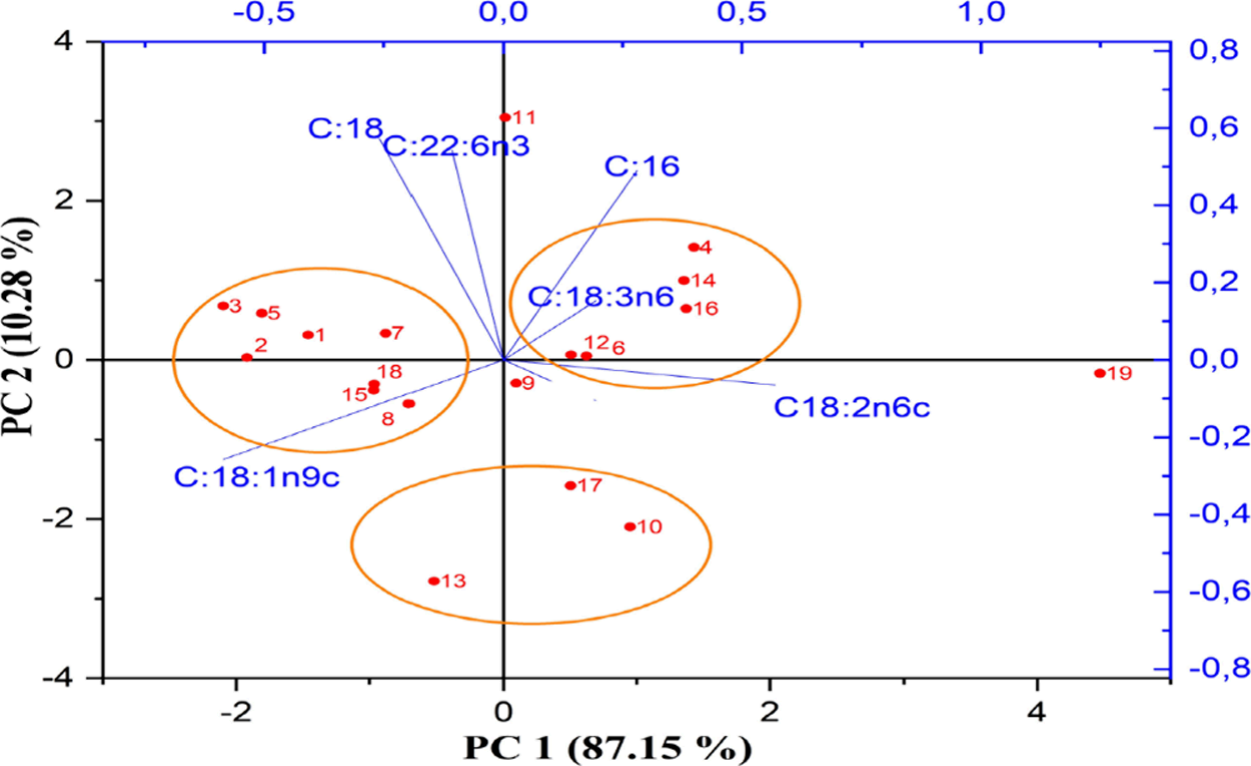
Comparison of the fatty acid content of SCOs by principal component analysis.

The PCA data indicate that the majority of the samples were distributed along PC1, suggesting significant differences between the samples. The variables such as C:18:3n6, C:18:2n6c, and C:16 were strongly aligned along the PC1 axis and exhibited a positive relationship. The FA composition of trial runs 4, 6, 12, 14, and 16 showed similarities in their C:18:3n6, C:18:2n6c, and C:16 contents. Similarly, palmitic acid (C:16:0), stearic acid (C:18:0), and γ-linolenic acid (C:22:6n3) demonstrated positive relationships with respect to PC2.

SCO production allows for the customization of fat profiles with the generation of oils with specific fatty acid compositions (MUFAs or PUFAs).^72–74^ SCOs therefore offer a promising alternative to both vegetable-derived oils and saturated fats in the food industry.^72–74^ The separation of the samples in the PCA (Figure 3) suggests that optimization of fermentation conditions can be carried forward depending on the kind of oil content that is desired.

### Chemical Properities of Single-Cell Oils

Edible oils are prone to oxidation during processing and storage, especially in the presence of high temperature, light, oxygen, and metal ions. The peroxide value (PV) is a vital indicator of the initial stages of oxidation and is generally used to monitor lipid oxidation during oil processing and preservation.^75^ Mathematical modeling conducted in the current study indicated that the PV of the oil samples from the 20 test runs ranged between 9.10 ± 0.06 and 10.25 ± 0.07 MeqO_2_/kg, while the free fatty acid (FFA) content ranged between 0.32 ± 0.06 and 0.48 ± 0.15 mg of KOH/g (Table 7). Among the 20 test runs, 6, 7, 8, 11, 15, and 20 exhibited identical parameters (pH, substrate amount, and fermentation duration), resulting in no statistically significant differences in their chemical properties. For this reason, data for the trial runs 6, 7, 8, 11, and 15 were excluded from Table 7. Additionally, in run 19, the fermentation duration was only 1.05 days, which was insufficient to achieve adequate biomass production. Consequently, oil production did not occur, and functional characterization could not be carried out. The FFA content is a quality indicator of the fat produced. A higher FFA level is associated with lower oil quality as it leads to bitterness and is also harmful for health.^76^ Single-cell fats contain significant amounts of natural antioxidants that protect FAs from oxidation and make them less vulnerable to oxidation compared to fats from plants and marine animals.^71^ Microbial oil produced from whey had 1.61% (KOH/g) of FFA, while the peroxide value was 20.99 (MeqO_2_/kg).^77^

**Table 7:** Chemical Properities, Bioactive Compounds, and Antimicrobial Activity of SCOs^a^

**run**	**1**	**2**	**3**	**4**	**5**	**9**	**10**	**12**	**13**	**14**	**16**	**17**	**18**	**19**	**20**
DPPH-IC_50_ (mg L^–1^)	48.55 ± 5.92^bc^	35.51 ± 1.30^ef^	nd.	38.46 ± 0.08^de^	nd.	nd.	nd.	36.63 ± 1.93^ef^	58.55 ± 1.87^a^	52.48 ± 1.67^ab^	44.73 ± 5.70^cd^	31.25 ± 0.62^f^	56.95 ± 1.50^a^	nd.	24.04 ± 0.07^g^
CUPRAC (μmol TE/kg extract)	17.92 ± 0.24^d^	19.99 ± 0.25^cd^	0.12 ± 0.03^f^	18.51 ± 0.88^cd^	0.15 ± 0.01^f^	0.20 ± 0.01^f^	0.81 ± 0.00^f^	19.35 ± 1.97^cd^	13.37 ± 0.63^e^	15.03 ± 1.69^e^	18.25 ± 1.23^cd^	27.48 ± 2.20^b^	14.22 ± 0.17^e^	nd.	30.43 ± 0.90^a^
total phenolic (mg GAE/kg extract)	359.83 ± 38.57^cd^	329.40 ± 21.00^de^	0.88 ± 0.19^g^	353.40 ± 3.86^cd^	1.26 ± 0.18^g^	1.40 ± 0.05^g^	1.86 ± 0.13^g^	392.83 ± 15.00^c^	601.11 ± 3.00^b^	289.54 ± 5.14^ef^	268.97 ± 19.71^f^	659.40 ± 66.43^a^	608.40 ± 1.71^ab^	nd.	594.6 ± 3.75^b^
peroxide value (MeqO_2_/kg)	9.50 ± 0.02^abc^	9.72 ± 0.04^abc^	10.09 ± 0.024^ab^	9.74 ± 0.15^abc^	10.15 ± 0.13^a^	10.12 ± 0.17^ab^	10.25 ± 0.07^a^	9.45 ± 0.45^abc^	9.72 ± 0.18^abc^	9.86 ± 0.34^abc^	9.25 ± 0.43^c^	9.10 ± 0.06^abc^	9.31 ± 0.43^bc^	nd.	9.75 ± 0.48^abc^
free fatty acid (mg KOH/g)	0.32 ± 0.06^ab^	0.34 ± 0.13^ab^	0.45 ± 0.12^a^	0.36 ± 0.07^a^	0.48 ± 0.15^a^	0.46 ± 0.16^a^	0.45 ± 0.15^a^	0.38 ± 0.12^a^	0.36 ± 0.13^a^	0.34 ± 0.15^ab^	0.38 ± 0.04^a^	0.36 ± 0.13^ab^	0.35 ± 0.11^a^	nd.	0.32 ± 0.21^ab^
Staphylococcus aureus ATCC 25923	1.17 ± 0.32^Ca^	4.03 ± 0.20^Ab^	<0.01	<0.01	<0.01	<0.01	<0.01	<0.01	3.12 ± 0.05^Bc^	0.81 ± 0.42^Cb^	<0.01	6.50 ± 0.40^Aa^	<0.01	<0.01	4.20 ± 0.08^Ab^
Listeria monocytogenes ATCC 7644	<0.01	5.98 ± 0.17^Aa^	<0.01	<0.01	<0.01	<0.01	<0.01	<0.01	3.24 ± 0.02^*Cc*^	<0.01	5.62 ± 0.195^Bb^	6.52 ± 0.35^Aa^	<0.01	<0.01	6.03 ± 0.13^Aa^
Salmonella enterica subs.ATCC 13076	<0.01	6.09 ± 0.08^Ba^	<0.01	1.46 ± 0.04^Ea^	<0.01	<0.01	<0.01	2.68 ± 0.07^Da^	5.31 ± 0.48^Ca^	0.72 ± 0.04^Fb^	11.12 ± 0.02^Aa^	6.25 ± 0.30^Ba^	<0.01	<0.01	6.03 ± 0.23^Ba^
Escherichia coli O157:H7 ATCC 33150	<0.01	6.13 ± 0.03^Aa^	<0.01	<0.01	<0.01	<0.01	<0.01	<0.01	3.98 ± 0.08^Bb^	1.96 ± 0.06^Ca^	<0.01	6.48 ± 0.36^Aa^	<0.01	<0.01	6.11 ± 0.01^Aa^

a ^a–g^ represent statistically difference between samples (*p* < 0.05). Run 20 represents runs 6, 7, 8, 11, and 15 samples produced under the same fermentation conditions. The antimicrobial activity is represented by the zone diameter in mm.

### Bioactive Compounds of Single-Cell Oils

Table 7 presents a comparison of the bioactive components in oil samples obtained under various fermentation conditions. The DPPH-IC_50_ values could not be determined for runs 3, 5, 9, and 10; however, the TAC value could be determined and was found to be 18–250 times lower than those of the other samples. Total phenolic content was found to be the lowest in runs 3, 5, 9, and 10, which was corroborated by their low antioxidant activity. The IC_50_ value indicates the concentration of DPPH required for 50% inhibition; therefore, lower values are considered to be more favorable. The samples showing the highest antioxidant activity in descending order were 20, 17, 2, 12, and 16. However, the highest phenolic content was detected in sample 17, followed by samples 13, 18, and 20; the latter three samples had similar phenolic contents. The samples with the highest concentrations of bioactive components were 20 and 17. The antioxidant effects of SCO may be due to the presence of phenolic hydroxyl groups in their structural components.^78,79^

Rhodotorula mucilaginosa is known to produce carotenoids and lipids.^80^ The total carotenoid contents of SCOs showed a statistically significant difference between the different samples (*p* < 0.05). The lowest carotenoid content of 2.64 ± 0.60 mg/100 g was found in run 1 (fermentation duration 5 days, carbon ratio 11.26%, and pH 4.75), while the highest carotenoid content was 54.90 ± 0.01 mg/100 g in run 17 (fermentation duration 8 days, carbon content 2.00%, and pH 6.00). This was followed by runs 16, 13, and 20 (Figure 4a), showing good agreement with their antioxidant content. Thus, samples with a high carotenoid content were generally fermented for longer periods of time (8 days), while the carbon content and pH varied.

**Figure 4:**
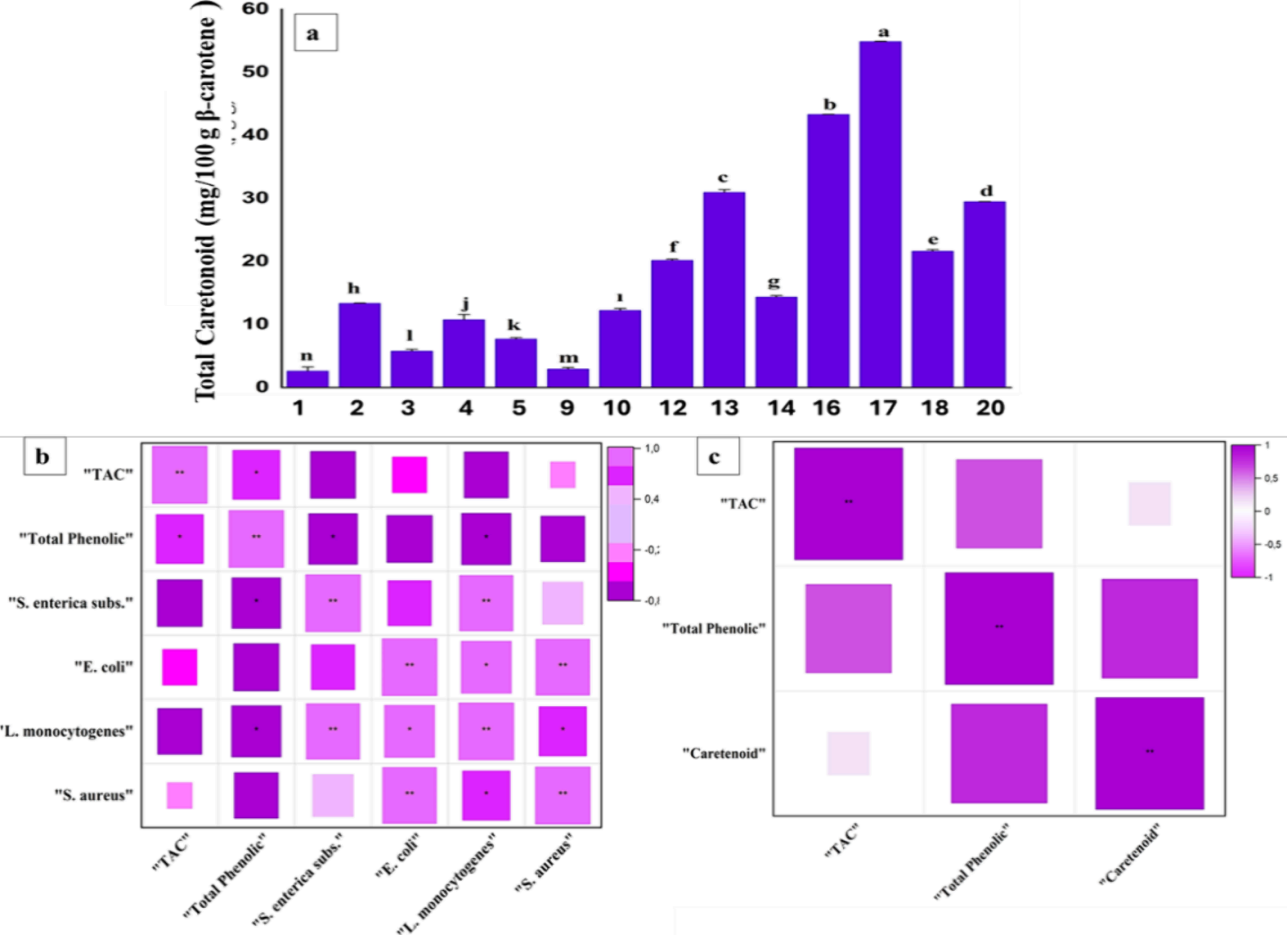
(a) Total carotenoid content mg/100 g β-carotene of single cell oil samples. (b, c) Correlation between TAC, total phenolic content, and antibacterial activity of SCOs against four different microorganisms (* *p* < 0.05, ** *p* < 0.01). ^a–g^ represent statistically difference between samples (*p* < 0.05).

Due to their unsaturated structure, carotenoids have a strong antioxidant activity that can prevent the oxidation of low-density lipoproteins and can also support a strong immune system.^81^ Microbial fermentation can lead to the formation of a considerable amount of natural carotenoids and phenolic compounds.^82^ Rhodotorula mucilaginosa offers significant advantages over bacteria, microalgae and plants in the production of carotenoids due to its unicellular structure, rapid proliferation ability, ability to grow on a wide range of substrates, and easy cultivation in large-scale fermenters.^83^

In a study investigating the biotechnological production of bioactive compounds using Rhodotorula mucilaginosa and acerola (Malpighia emarginata L.) seeds,^83^ the amount of phenolic compounds in the biomass was similar to the observations in the current study. The phenolic components of SCOs may have been synthesized during fermentation or may have been incorporated into the structure from grape pomace, which is also known to be rich in phenolic substances^84^

The concurrent synthesis of carotenoids and fatty acids by Rhodotorula mucilaginosa can augment the nutritional and physiological efficacy of SCOs,^85,12^ suggested that preferential accumulation of oleic acid and PUFAs in the SCOs instead of SFA would make the production of SCOs containing both lipids and carotenoids from red oil yeasts more economical. The SCOs produced in this study meet these requirements. Additionally, it is very likley that the high β-carotene content of Rhodotorula mucilaginosa BT59 ^86^ may contribute toward the antioxidant properties of the SCOs generated. Therefore, we were successful in generating a high-value-added oil that can be used in the food industry, is beneficial to health, and is resistant to oxidation.

### Antimicrobial Activity of SCOs

The SCOs generated in this study were also tested for their antimicrobial activity against various human pathogens (Table 7). Runs 2, 16, 17, and 20 showed the highest antimicrobial properties with the largest zone diameters. These samples contained palmitic, palmitoleic, stearic, and γ- linoleic acid in similar proportions, indicating that oils with strong antimicrobial properties had been produced. In particular, run 16 showed the highest antimicrobial activity toward Salmonella enterica subs. ATCC 13076. Runs 17, 20, and 2 demonstrated antimicrobial activity against all pathogens tested. Moreover, run 17 exhibited the highest antimicrobial activity against all pathogens except Salmonella enterica subs. Runs 3, 5, 9, 10, 18, and 19 did not demonstrate any antimicrobial activity against the pathogens tested. Although PUFAs with antimicrobial and/or anticancer activities are found in various foods, commercially available sources are very limited but can be synthesized from microbial sources such as certain strains of yeast and algae.^87–90^ Antibiotic resistance represents a major challenge in healthcare, leading to the necessity of discovering and using new molecules such as essential oils,^91–93^ further highlighting the importance of optimal SCO production from Rhodotorula mucilaginosa BT59.

### Correlation Analysis

A Pearson correlation analysis was carried out to determine the relationship between TAC, phenolic content, and antimicrobial activity of SCOs. We observed that the total phenolic content was highly correlated with TAC (*r* = 0.74, *p* < 0.05), antimicrobial activity against L. monocytogenes (*r* = 0.81, *p* < 0.05), as well as S. enterica subs. (*r* = 0.74, *p* < 0.01) (Figure 4b). Phenolic compounds have previously been shown to have antimicrobial activities against L. monocytogenes.^94–96^ We also observed a high positive correlation between S. enterica subs. and L. monocytogenes (*r* = 0.95, *p* < 0.01), and between E. coli and S. aureus (*r* = 0.96, *p* < 0.01). The total phenolic content had a significant positive relationship with TAC (*r* = 0.63, *p* < 0.05) and total carotenoid content (*r* = 0.78, *p* < 0.05) (Figure 4c). Similar correlations between antioxidant activity, total phenolic, and carotenoid contents have been reported previously.^97,98^ The high phenolic content as well as antioxidant and antimicrobial activities of SCOs increase their potential as high-value oil alternatives.

## Conclusions

Single-cell oil (SCO) from Rhodotorula mucilaginosa BT59 was produced using grape pomace, a sustainable and cost-effective raw material, as the substrate. These oils can be considered as potential sources of lipids enriched in phenolic compounds and carotenoids. Optimization studies revealed that SCO with a high unsaturated fat content was best achieved with fermentation for 8 days, 2% carbon content, and pH 4.47, while high saturated fat content required fermentation for 8 days, 8.32% carbon content, and pH 4.44. Variations in the FA profile as a function of the fermentation conditions may make this product a viable alternative to oils commonly used in industry such as olive oil, palm oil, and cocoa butter. Additionally, SCO was evaluated for its bioactive components and antimicrobial properties. Fermentation conditions of 8 days, 2% carbon content, and pH 6.00 yielded the highest levels of bioactive compounds and antimicrobial activity, followed by fermentation of 5 days, 6% carbon content, and pH 4.75. The strong correlation between the functional properties of the SCO underscores the reliability of our findings and provides a foundation for tailoring SCO production to meet specific industrial or nutritional requirements. Overall, our study suggests that SCO derived from Rhodotorula mucilaginosa BT59 demonstrates significant potential as a sustainable lipid source. Its customizable fatty acid profiles and bioactive properties make it an economically viable and environmentally friendly alternative to conventional oils, offering opportunities for broad applications across various industries.
